# Sequence-dependent response of DNA to torsional stress: a potential biological regulation mechanism

**DOI:** 10.1093/nar/gkx1270

**Published:** 2017-12-18

**Authors:** Anna Reymer, Krystyna Zakrzewska, Richard Lavery

**Affiliations:** 1 Department of Chemistry and Molecular Biology, University of Gothenburg, Gothenburg 40530, Sweden; 2 Institut de Biologie et Chimie des Protéines, Université de Lyon I/CNRS UMR 5086, Lyon 69367, France

## Abstract

Torsional restraints on DNA change in time and space during the life of the cell and are an integral part of processes such as gene expression, DNA repair and packaging. The mechanical behavior of DNA under torsional stress has been studied on a mesoscopic scale, but little is known concerning its response at the level of individual base pairs and the effects of base pair composition. To answer this question, we have developed a geometrical restraint that can accurately control the total twist of a DNA segment during all-atom molecular dynamics simulations. By applying this restraint to four different DNA oligomers, we are able to show that DNA responds to both under- and overtwisting in a very heterogeneous manner. Certain base pair steps, in specific sequence environments, are able to absorb most of the torsional stress, leaving other steps close to their relaxed conformation. This heterogeneity also affects the local torsional modulus of DNA. These findings suggest that modifying torsional stress on DNA could act as a modulator for protein binding via the heterogeneous changes in local DNA structure.

## INTRODUCTION

Understanding how the underlying organization of genome contributes to biological regulation is an important question. One important element in this organization is linked to the torsional strain that is imposed on the DNA double helix by many essential biological processes. This strain leads to supercoiling, namely axial bending combined with over- or undertwisting, and potentially to the formation of interwound plectonemic structures. One example of such a process is transcription where immobilized RNA polymerase within transcription factories forces DNA to rotate around its axis as the double helix threads through the transcription machinery (1,2). This leads to undertwisting upstream DNA (negative supercoiling) and overtwisting downstream DNA (positive supercoiling). These changes in supercoiling can then propagate along a chromatin fiber, with ranges and speeds of propagation that are dependent on the underlying nucleotide sequence (3). Modifying supercoiling will impact chromatin structure at many scales, locally modifying DNA conformation (notably, bending and twisting), stabilizing or destabilizing nucleosome core particles (4), and changing higher-order chromatin structures, with a resultant impact on protein–DNA interactions (5).

*In vivo* studies, using various DNA-mapping probes, show that the overall bulk of genomic DNA in mammalian cells is torsionally relaxed, while supercoiling exists at some loci (6,7). More recent investigations by Naughton *et al.* showed that genomes are organized into supercoiling domains (3). These domains are delimited by CG/AT boundaries, but are constantly remodeled by cellular machines. The CG-rich domains are, on average, significantly negatively supercoiled, highly transcribed, and enriched in open chromatin fibers, transcription initiation sites, RNA polymerase and topoisomerase I binding sites. In view of these observations, a comprehensive understanding of the sequence-dependent energetics and mechanics of DNA under torsional strain is important.

Despite being a crucial factor for chromosomal structure and function, little is known about the local structure of torsionally constrained DNA. Experimentally, supercoiling is studied using ensemble-average techniques, such as electron microscopy or electrophoretic separation (8,9), or single-molecule manipulation techniques, such as magnetic tweezers (10,11). While the first group of techniques probe overall supercoiling-mediated changes in compaction and overall geometry, the second category can provide dynamic information, for example, by monitoring supercoiling-diffusion, or the behavior of nucleosome arrays (12–14). These techniques provide valuable insights into the mechanical properties of DNA on the mesoscale, but little information on sequence-dependent structural rearrangements that may have important biological consequences, not least for the formation of regulatory protein–DNA complexes.

The mechanics of DNA supercoiling has also been addressed by various theoretical approaches. A number of phenomenological models of different complexity have been developed, including the worm-like-chain and rod-like-chain approaches (15–18) that describe DNA as an isotropic elastic polymer with constant persistence length, diameter and charge density. However, these models are limited to a description of the overall mechanical response of sufficiently long DNA molecules, typically hundreds to thousands of base pairs (bp) under moderate torsional stress (10,19).

Moving toward an atomistic view of DNA deformations, Olson *et al.* addressed the local plasticity of the double helix by analyzing structural fluctuations in crystal structures of protein–DNA complexes (20) and derived a set of empirical energy functions for deformations of the double helix. More recently, progress in all-atom molecular dynamics (MD), and in nucleic acids force fields, has enabled more detailed studies of torsionally stressed DNA. Using a simple twist restraint and relatively short MD sampling, Kannan *et al.* were able to generate free energy profiles with respect to twist and found evidence for sequence-dependent response to torsional stress, with pyrimidine(Y)-purine(R) (also denoted YpR) steps showing the highest response to under- and overtwisting (21). Pettitt and co-workers (22) also exposed the double helix to extreme torsional stresses, while restricting bending, and reported spontaneous sequence-dependent base flipping upon underwinding and the formation of Pauling-like DNA structures (23) upon overwinding. Lastly, Harris and co-workers explored the conformational space of supercoiled DNA minicircles and also observed deformations of the double helix, including the formation of single-stranded bubbles and ‘wrinkles’, induced by undertwisting (24). These studies provide valuable insights, but have not addressed sequence-dependent effects in detail. Other authors have used coarse-grain DNA models including sequence effects, but these assume that base pair steps deform harmonically and thus do not explicitly treat transitions between local conformational substates (25,26).

That such substates are important became clear during recent and comprehensive studies of the sequence-dependent conformational dynamics of DNA by the laboratories belonging to the ABC consortium (27,28). These studies have shown that certain dinucleotide steps are bimodal and can spontaneously adopt two different conformations. Helical twist is one of the variables involved, and it was found that YpR steps are particularly prone to twist bimodality (with substates typically separated by roughly 20° in twist). In addition, some purine(R)-purine(R) (denoted RpR) steps also show twist bimodality, but with a somewhat smaller range of twists. However, it was also noted that the sequences flanking such steps can significantly modify the degree of bimodality and also the preference for a low or high twist state. It is clear that such behavior may play an important role in how DNA reacts to torsional strain and the aim of this study is to address this question.

In order to do this, we have developed a new structural constraint, based on the Curves+ conformational analysis methodology (29), which controls the total helical twist between two chosen base pairs, allowing both under- and overtwisting to be imposed. This restraint has been implemented within PLUMED free energy library (30) and can thus be used in conjunction with a variety of standard all-atom MD software packages. While determining the total twist of a chosen segment of DNA, our restraint has no influence on other helical variables and it does not control how a segment will react locally to torsional stress. While a single restraint is typically applicable to a few turns of the double helix, several restraints can be chained together to study twisting in longer DNA fragments. The restraint can be applied to DNA alone and or to DNA complexes with other molecules.

As a preliminary test of how DNA responds to torsional stress in a sequence-dependent manner, we present results on four 17 bp oligomers (see Table 1), whose central segments contain repeat sequences involving the tetranucleotides ACGT, ACGA, CCGA and AGCT. Using the latest Parmbsc1 force field (31) coupled with our new restraint, we now investigate how these oligomers respond to over- and undertwisting. The results show that torsionally stressed DNA is highly heterogeneous. Sequence-dependence plays a significant role and leads to the notion of ‘twist capacitor’ dinucleotides that, in a given flanking sequence-environment can locally store and release torsional stress.

**Table 1.** DNA oligomers studied, showing the four-letter code used to refer to them and the presence of potentially bimodal YpR and RpR steps

Oligomer	Sequence (5′→3′)	Restrained YpR steps	Restrained RpR steps
ACGT	GC**ACGTACGTACGTA**GC	R**CpG**Y, R**TpA**Y, (R**TpA**R)	
ACGA	GC**ACGAACGAACGAA**GC	R**CpG**R	R**ApA**Y,(R**ApA**R)
CCGA	GC**CCGACCGACCGAC**GC	Y**CpG**R	Y**GpG**Y
AGCT	GC**AGCTAGCTAGCTA**GC	Y**TpA**R	Y**ApG**Y

The twist restraint is applied to the bold and underlined segment of each oligomer. YpR and RpR steps in brackets occur at the end of the restrained segment and have different flanking base pairs.

## MATERIALS AND METHODS

### Restraining the helical twist

The helical twist of DNA should be measured around the helical axis, which cannot always be assumed to be a straight line. To take this problem into account we follow the general approach adopted by Curves+ (29). This requires defining local helical axes *U_i_* and *U_j_* at either end of the segment to be restrained. In order to obtain the *U* vectors, we begin by creating a local frame for each nucleotide **b** whose three unit vectors are derived from atoms belonging to each base: (i) the bond vectors N9-C1′ for purines, or N1–C1′ for pyrimidines; (ii) the cross product between this vector and the difference vector C8-C1′ for purines, or C6-C1′ for pyrimidines; (iii) the cross product between the two former vectors. The rotation matrix between two successive base frames can be obtained as **Q_12_** = **b_1_^T^b_2_. Q** is a 3 × 3 rotation matrix associated with three eigenvectors and three eigenvalues. The first eigenvector defines the *U_12_* screw axis linking the two nucleotide frames by a rotation θ_12_ around the axis and a translation λ_12_ along the axis, where Cos θ_12_ = [trace(**Q_12_**) - 1]/2.

The local helical axis *U_i_* is then obtained by averaging the inter-base screw axes *U_i-1,i_* and *U_i,i+1_* in each strand of the double helix (i.e. four unit vectors in total). *U_j_* is similarly the average of *U_j-1,j_* and *U_j,j+1_* within each strand. By combining *U_i_* (and similarly *U_j_*) with the unit vector along the N1-N9 axis of base pair *i* (oriented in the direction from the 5′-3′ to the 3′-5′ strand), we are able to create an axis frame **B_i_** (and similarly **B_j_**) associated with each end of the restrained DNA fragment. Solving the equation **Q_ij_** = **B_i_^T^B_j_** provides the total rotation between the two axis frames θ_ij_ in the same manner described for the inter-base frames above. Finally, the total twist (the component of the total rotation corresponding to rotation around the helical axis) between **B_i_** and **B_j_** is given by *twist* = θ_ij_(*U_ij_·U_i_*) where *U_ij_* is now the screw vector relating the **B_i_** and **B_j_** frames.

During the MD simulation, we can restrain the twist between base pairs *i* and *j* to the desired value *twist_ref_* using a simple quadratic function, *k*(*twist-twist_ref_*)^2^. Following initial trials, the force constant *k* was chosen as 0.06 kcal mol^–1^ degrees^–2^, the smallest value which was able to achieve the desired twist. Note that since θ_ij_ is only defined in the range ±180°, the twist restraint can only be applied to oligomeric fragments of DNA, typically a few turns, where, in addition, the limited curvature implies that the restrained twist will remain close to that measured with the curvilinear axis derived by Curves+. Initial trials showed this to be true to within a few tenths of a degree. If it is necessary to control the total twist of longer DNA fragments, several restraints acting on short segments can be chained together.

The new twist restraint was implemented within version 2.2.1 of the PLUMED free energy library environment (30). This allows it to be used in combination with a variety of MD software packages. The C++ code and a user guide for the twisting constraint can be found here: https://github.com/annareym/PLUMED_DNA-Twist.

### Over- and undertwisting DNA oligomers

For each of the 17-mer oligomers studied, the twisting restraint was applied between the 3rd and 15th bp (i.e. 12 bp steps, see Table 1). This avoids potential problems linked to deformations within the weaker base pairs close to the oligomer termini. The total twist between these base pairs was increased or decreased in 6° steps (corresponding to an average change in twist of 0.5° per base pair step. Following MD simulation of each step (see below), convergence was improved by using the final structure under each twist restraint as the starting structure for the following step, working outward in the over- and undertwisting directions from the relaxed oligomer. Over- and undertwisting of each oligomer was limited to a maximum of ±5° with respect to 34.9°, the relaxed twist per base pair step averaged over the four oligomers studied. Note that this range is approximately double that sampled by torsionally unrestrained DNA at room temperature.

### Molecular dynamics umbrella sampling simulations

The DNA oligomers studied were initially constructed in standard B-DNA conformations using the nucleic acid modeling program JUMNA (32). Unrestrained MD simulations followed by umbrella sampling simulations were both performed using the GROMACS MD software package, version 5.1 (33). Simulations were carried out using the latest AMBER all-atom nucleic acid force field Parmbsc1 (31). Note that this parameter set avoids artefacts that, after a few tens of nanoseconds of simulation, caused a build-up of unusual backbone conformations involving the α (P-O5′) and γ (C5′-C4′) dihedrals and resulted in a steady loss of helical twist. The parmbsc1 force field has been the subject of extensive testing and has been shown to accurately reproduce a wide range of experimental data on DNA (31).

Each oligomer was first neutralized with 32 K+ counterions and solvated with TIP3P water molecules (34) corresponding to a solvent layer of 10 Å, contained within a cubic cell under periodic boundary conditions. Additional K^+^ and Cl^−^ ions were then added to achieve a physiological salt concentration of 150 mM. The conformation of each oligomer was initially energy minimized with 5000 steps of steepest descent, followed by a 200 ps simulation at constant volume, while raising the temperature to 300 K. Simulations were then carried out at constant pressure and temperature (1 atm., 300 K) using a weak-coupling thermostat (35) with a 0.2 ps coupling constant and an isotropic Parrinello-Rahman barostat (36) with a 2 ps coupling constant. Simulations used a 2 fs time step. Bonds involving hydrogen atoms were constrained with the LINCS algorithm (37) and the non-bonded pair list was updated every 20 fs with the group scheme (38). Electrostatic forces were evaluated with particle-mesh Ewald (39) with a real-space cutoff of 10 Å. The van der Waals forces were truncated at 10 Å and long-range corrections were added. Center of mass movement was removed every 0.2 ps to avoid the building up translational kinetic energy (40).

Since restraints are necessary to study a sufficiently large range of helical twists, it is not possible to directly obtain free energy curves using the inverse Boltzmann procedure that determines the energy of a given state on the basis of its probability of occurrence. It is first necessary to correct the probabilities for the impact of the restraining potential. This can be done in an iterative manner using the so-called Weighted Histogram Analysis Method (WHAM) method (41). We used the version of this approach implemented in PLUMED (30) to calculate the corresponding potential of mean force (PMF) with respect to DNA twisting. Following a 300 ns simulation on each unrestrained oligomer, umbrella sampling was carried out with 100 ns of sampling per window. The initial 20 ns of the sampling time was discarded as equilibration and WHAM analysis was applied to two equal 40 ns blocks of data to test convergence. Overall the deviations between the PMF profiles representing the two sampling blocks were negligible (with maximum deviation of 0.02 kcal mol^−1^), with the exception of the AGCT oligomer for which sampling was extended to 150 ns per window to reach similar level of convergence. The total simulation time therefore was 2.4 μs for ACGT, ACGA and CCGA and 3.4 μs for AGCT.

### Conformational analysis

The conformational analysis of the recorded trajectories was performed in several stages, beginning with pre-processing using the cpptraj program (42) of the AMBERTOOLS 14 package. For each MD snapshot, extracted at 1 ps intervals, DNA helical parameters, backbone torsional angles, and groove geometry parameters were analyzed using Curves+ and Canal (29) (available at http://curvesplus.bsc.es), providing complete, time-dependent information on the response of the DNA oligomers to the imposed torsional stress.

## RESULTS AND DISCUSSION

The four oligomers studied in this work are listed in Table 1. These oligomers were chosen so as to include pyrimidine-p-purine (YpR) and purine-p-purine (RpR) steps that have already been shown to be able to exist in high and low twist states (28,43,44). Table 1 lists the presence of these steps within the segment of each oligomer that will be subjected to twist restraints (namely, base pairs 3–15). We also indicate the purine/pyrimidine (R/Y) nature of the flanking base pairs. Note that the restrained segments also contain 7 out of the 10 distinct dinucleotide steps, including two out of the three YpR steps and all four RpR steps.

**Figure 1.**
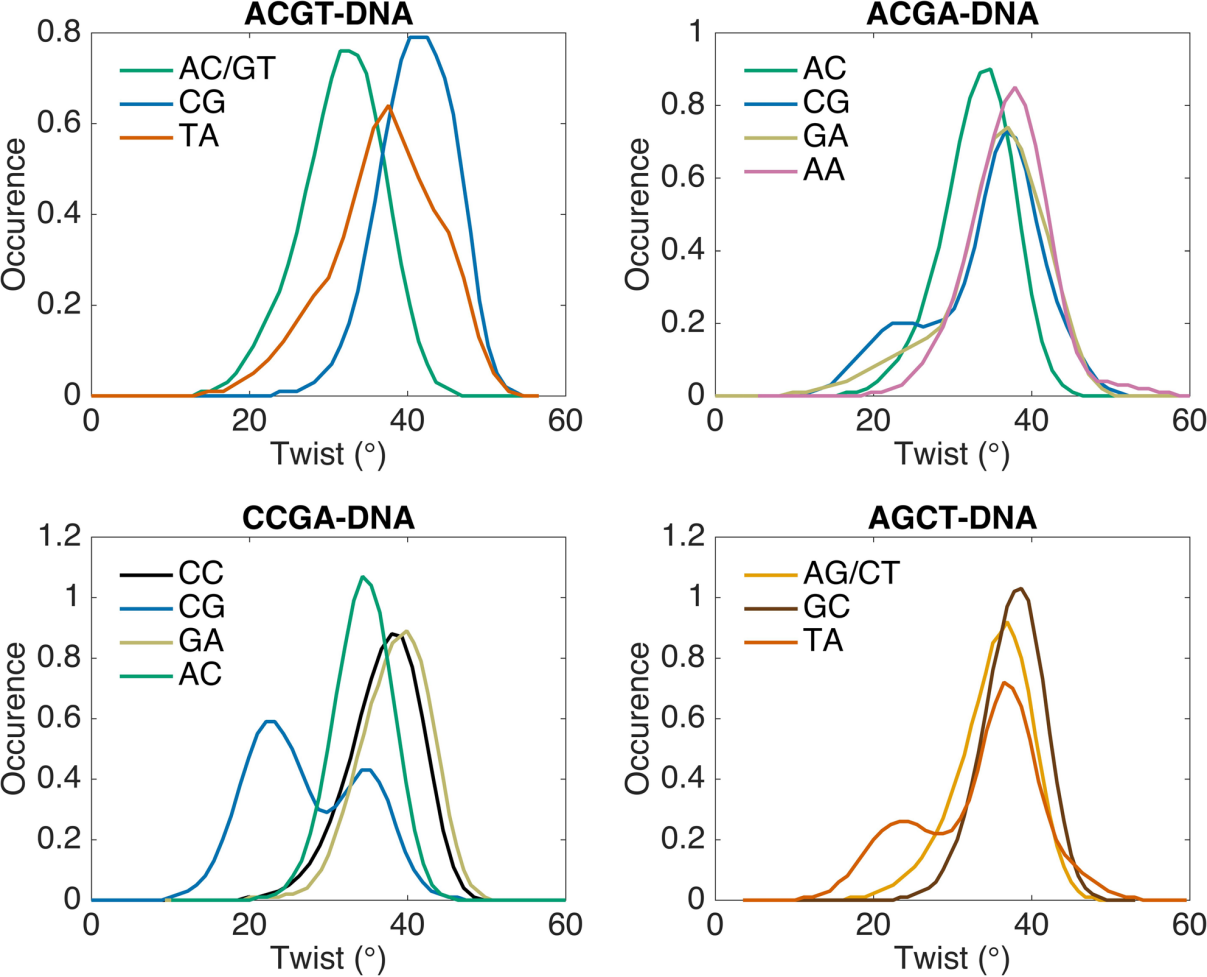
Twist distributions of dinucleotide steps derived from 300 ns unrestrained MD trajectories of the four oligomers.

The first three oligomers contain the YpR step CpG. The first and the fourth oligomers contain another YpR step, TpA. The last three oligomers contain the RpR steps: ApA in ACGA, GpG in CCGA and ApG in AGCT. Based on microsecond-scale ABC and subsequent studies using the AMBER Parmbsc0 force field (28,45) the CpG step within ACGT would mainly be expected to prefer a high twist, that within ACGA should have an intermediate twist and that within CCGA should prefer a low twist. The TpA step in the AGCT oligomer (i.e. CTAG) should also prefer a low twist, while that in the ACGT oligomer (i.e. GTAC) should prefer a high twist. However, all these steps should be able to shift between lower and higher twist substates. The AA step in ACGA (i.e. GAAC), the GG step in CCGA (i.e. CGGT) and the AG step in AGCT (i.e. TAGC) should all prefer high twists. These observations are confirmed for the Parmbsc1 simulations on our oligomers, as shown by 300 ns unrestrained MD twist distributions shown in Figure 1 for the base pair steps belonging to the central tetranucleotides. the single exception is the TpA step in AGCT, which, although it has a clear low twist population, spends more time in the high twist state. It should however be pointed out that twist conformer distributions can be very slow to fully stabilize.

### Sequence-dependent torsional modulus of DNA

Following 300 ns of unrestrained MD, the new twist restraint was used to modify the total twist of the 12 central base pair steps by ±5° with respect to the average twist per base pair of the restrained segments (34.9°). This corresponds to a change in superhelical density σ of approximately ±0.15, estimating this value for a straight DNA fragment on the basis of the average relaxed total twist. The base pairs of the restrained segments remained intact during both over- and undertwisting, although occasional opening angles beyond 30° was observed in the most undertwisted state (generally for less than 1% of the trajectory and only reaching 4 to 6% for 3 bp). No significant bending was observed, with the bending probability distributions showing maxima of at most 5°, with most values below 2°.

**Figure 2.**
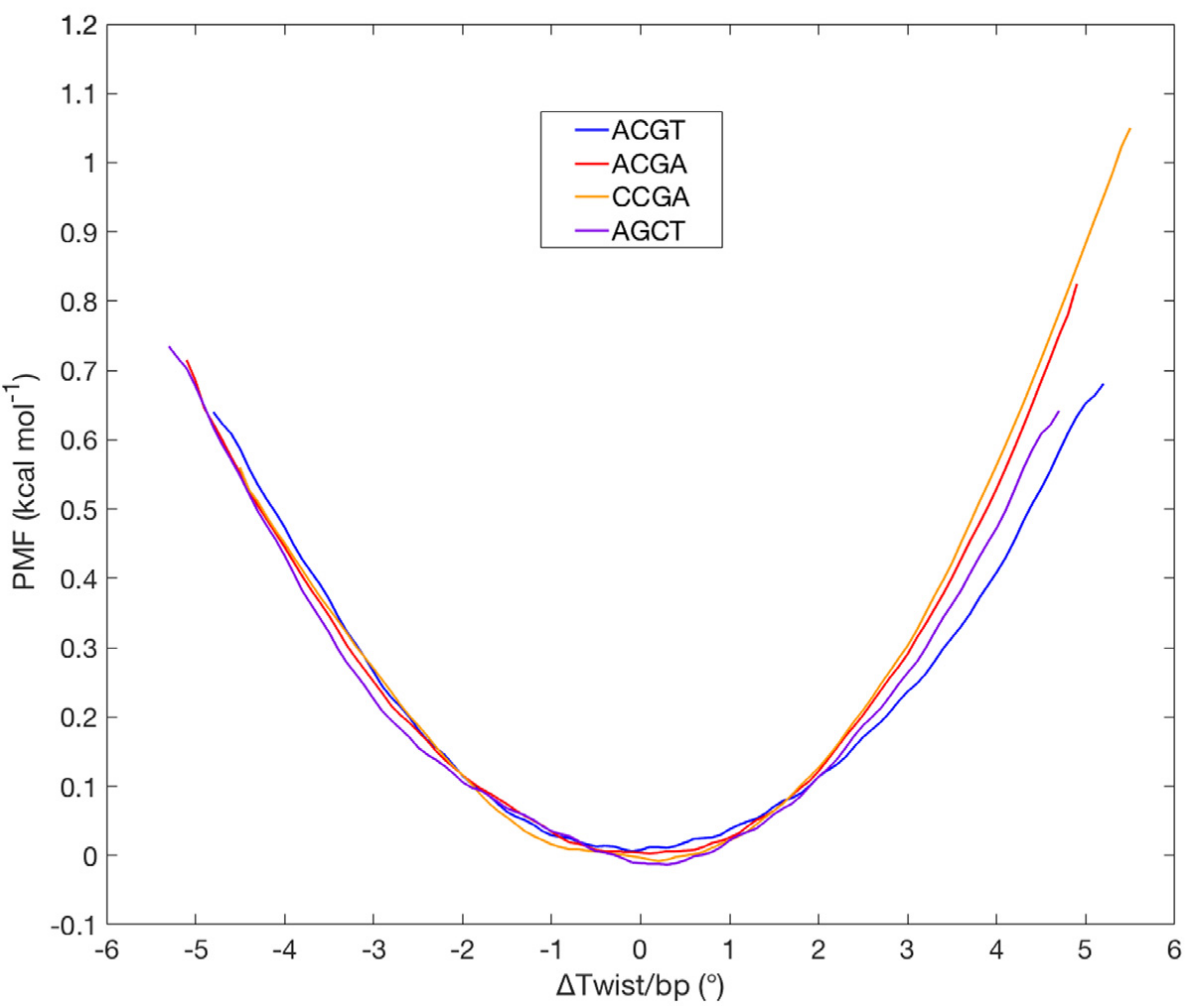
PMF with respect to the average change of twist per base pair step with respect to the corresponding value for the relaxed oligomer.

PMF plots, that describe the change in free energy as a function of the imposed twist change (shown in Figure 2), were obtained using the umbrella sampling protocol described above with 100 ns of sampling (150 ns for the AGCT oligomer) for each 6° change in total twist (corresponding to a 0.5° average twist change per base pair step). Although DNA is usually assumed to show a harmonic behavior with respect to small deformations, Figure 2 shows that the PMF curves are not truly symmetric about the minimum. We thus tried fitting the curves with both quadratic and cubic functions. In the case of ACGT and AGCT, the root mean square error (RMSE) was almost identical for quadratic and cubic fits (0.008 versus 0.007 kcal mol^−1^ for ACGT and 0.010 versus 0.008 kcal mol^−1^ for AGCT). However, ACGA and CCGA show somewhat more asymmetry around the minimum as evidenced by RMSE’s for quadratic and cubic fits of 0.015 versus 0.004 kcal mol^−1^ and 0.015 versus 0.007 kcal mol^−1^ respectively. In all cases, the curves show small irregularities around the minima where exchanges between several conformational substates occur frequently (see below). We have consequently derived the optimal average twist angles and the corresponding force constants from the fits to the overall PMF curves. In order to compare the results more easily, the curves are plotted as changes in the average twist per base pair step with the respect to the corresponding optimal twist value, which varies from 34.4° for CCGA to 35.2° for AGCT (see Table 2). The energies at the optimal twist values have also been shifted to zero. The results in Figure 2 show only a moderate sequence effect. On average, modifying a single base pair step by ±4° (which corresponds to change in superhelical density σ of approximately ±0.12) implies an energy cost of 0.45 kcal mol^−1^. We can also see from the figure that the asymmetry associated with ACGA and CCGA is due to a slightly higher resistance to overtwisting compared to undertwisting for these oligomers.

**Table 2.** Calculated average relaxed twists, torsional constants, torsional moduli and torsional persistence lengths for the oligomers studied.

Oligomer	<Relaxed Twist> (deg)	*K* (kcal mol^−1^/deg^2^)	*C* (pN nm^2^)	*P* (nm)
ACGT	34.7	0.054	420	102
ACGA	35.0	0.061	475	115
CCGA	34.4	0.064	495	120
AGCT	35.2	0.057	443	108

The force constants derived from the fitted curves (*K*) can be used obtain the torsional modulus *C*, using the isotropic rod equations, *T* = *K*Δθ = *C* Δθ*/L*, where *T* is the torque resulting from a change in twist Δθ over a length *L*. Note that we use a standard value of *L* = 0.34 nm in order to compare with other published results, although *L* is weakly sequence dependent and can also vary with the imposed torsion (see later). The torsional modulus can be used to derive the torsional persistence length *P* using the equation *P* = *C/k_B_T* (taking *k_B_T* at room temperature as 4.114 pN nm). The results are shown in Table 2. The resulting order of torsional stiffness is CCGA > ACGA > AGCT > ACGT. The calculated values are in the range of sequence-averaged experimental measurements using single molecule techniques (applying torsion while gently stretching the DNA preventing the formation of plectonemes) which yielded <*C*> = 410 ± 30 pN nm^2^ (implying <*P*> ≈ 100 nm) (46).

### Role of individual base pair steps in the response of DNA to torsional stress

**Figure 3.**
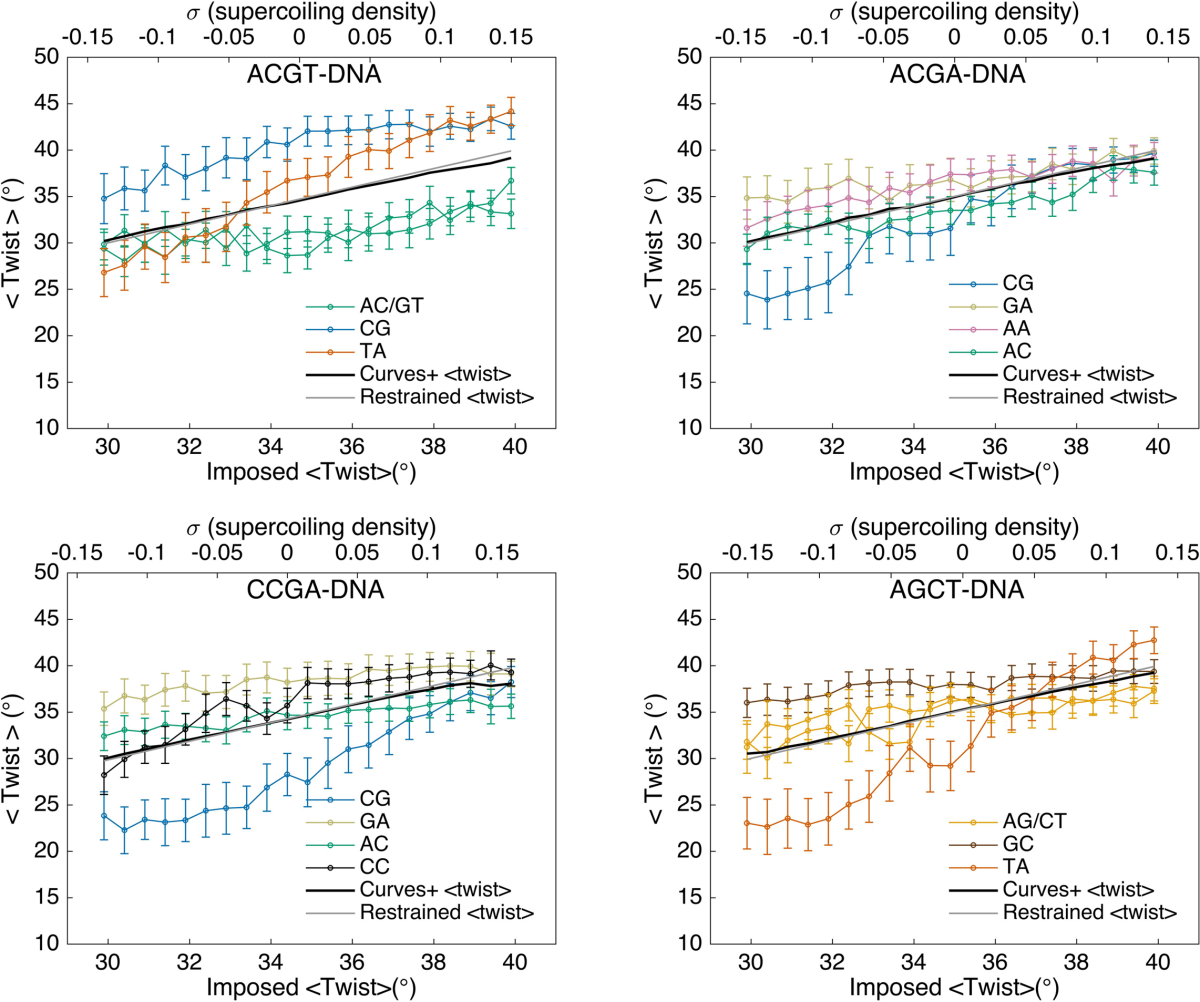
Twist response to the imposed restraint for the base pair steps constituting the central tetranucleotide of each of the four oligomers, showing average values (points) and standard deviations (vertical bars). In each plot, the black line shows the average twist over the 12-bp steps restrained region that can be compared with the desired twist imposed by our restraint (gray line).

In order to understand the detailed behavior of DNA with respect to changes in twist, we have to look at what happens to individual base pair steps as our twist restraint is applied. These data are presented in Figure 3. In passing, we remark that measuring the average twist over the restrained regions with conformational analysis program Curves+ leads to a value that is very close to the value imposed by our restraint confirming its validity.

If we now consider the behavior of individual base pair steps, we see that they do not respond equally to the imposed changes in twist. The most interesting steps are those whose slope deviates most from average twist per step, shown by the black line in each panel of Figure 3. For the first oligomer, ACGT, it is the TpA twist (yellow line) that shows a steeper decrease than the average during untwisting and a steeper increase during overtwisting. Since CpG steps can also convert between low and high twists (28,45), one might have expected the CpG step to act in a similar way. However, as noted above, an RCGY environment strongly favors a high twist (see Figure 1) and prevents its potential bimodality from being exploited in this case. Nevertheless, the adaptability of the TpA step relieves the stress on all the remaining steps, as seen in Figure 3, leading to twist slopes that are lower than the average. Each TpA step can effectively absorb up to 9° when the average undertwisting reaches −5°/bp step, and 7° when the average overtwisting reaches +5°/bp step.

In contrast to ACGT, the CpG steps of the ACGA and CCGA oligomers (blue lines in Figure 3) are able to move between low and high twist states (see also Figure 1). They consequently play an important role in absorbing the imposed twist, although the dominance of the intermediate twist state of CpG in the relaxed ACGA oligomer means that it is less efficient in absorbing overtwisting, with a maximum change of +4° versus a change of −10° with undertwisting. In the case of CCGA, the low twist state of the relaxed CpG step causes it to respond in the opposite manner, being most efficient at absorbing overtwisting stress, with a limiting change of +11° versus −5° with undertwisting. A consequence of this behavior is that undertwisting the CCGA oligomer brings the CpC/GpG steps into play. While they occupy the high twist state in the relaxed oligomer (see Figure 1), as expected for a YGpGY environment (28), they are able to move to a low twist state when the oligomer is undertwisted.

Lastly, for the AGCT oligomer Figure 3 shows that the TpA step again plays a major role in absorbing twist. This step absorbs 10° during maximum undertwisting, and 9° during maximum overtwisting. Although the potentially bimodal ApG step is also present in this oligomer it predominantly occupies the high twist state in the relaxed oligomer (see Figure 1) and resists changing this state.

As expected, the large twist changes for TpA steps in ACGT and AGCT are linked to coupled changes in the backbones. As seen in earlier work (27,28), these involve the phosphate linkages on the 3′-side of the YpR steps in both strands, with high twists being associated with BI states (ϵ/ζ trans/gauche-) and low twists with BII states (ϵ/ζ gauche-/*trans*). Supplementary Figure S1 confirms this behavior, with bimodality in the relaxed oligomers being replaced with a dominance of BII state upon undertwisting and preference for BI states upon overtwisting.

**Figure 4.**
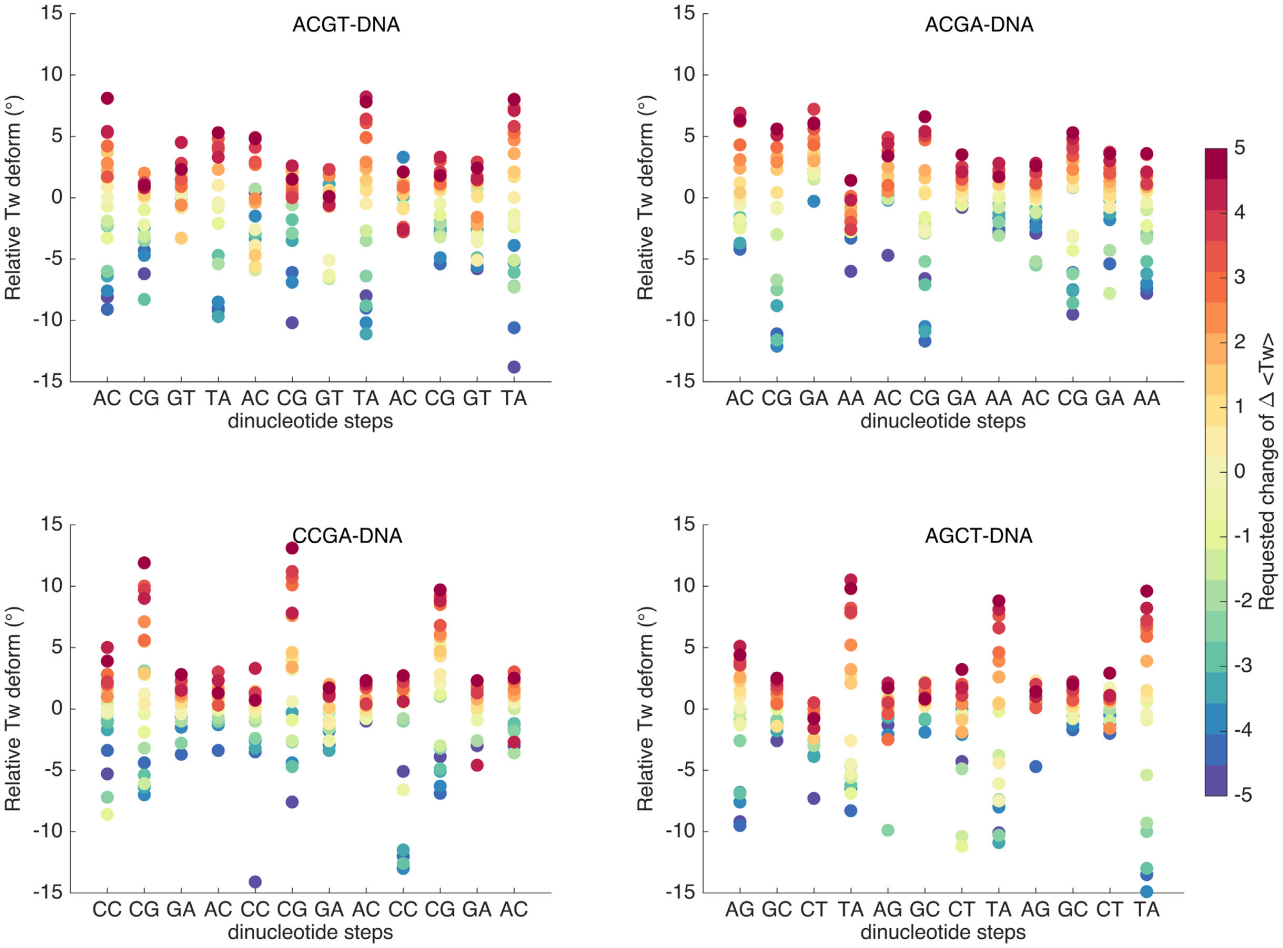
Changes of twist angles for the restrained segment of each oligomer with respect to their relaxed values as a function of the imposed average twist per base pair (Relative Tw deformation (°) = <Tw>^actual^ − (<Tw>^equilibrated^ − ΔTw^requested^)). The coloring of the dots indicates the imposed twist, red to blue corresponding to the range +5° (overtwisting) to −5° undertwisting with respect to the average base pair step twist of the restrained segments (34.9°).

Up to this point, we have limited our discussion to the central tetranucleotide steps of our oligomers. In fact, as shown in Figure 4, the same observations apply to all equivalent steps in the restrained portion of the oligomers. In this figure, the length of the vertical bars indicates the range of twists covered by each step as the oligomer is undertwisted and overtwisted. This figure however emphasizes the importance of the base pairs flanking each step. Thus, while RTpAY (namely, GTpAC) steps indeed absorb most of the twist in the ACGT oligomer, the 3′-TpA step (on the right of the plot) is in a different environment (GTpAG) and turns out to be even more adaptable to undertwisting. Similar changes in a single flanking base pair change the twist behavior of the 5′-ApC step of ACGT, the 3′-ApA step of ACGA and the 5′-ApG step of AGCT.

Summarizing these results, we can make two points. First, each oligomer we have studied contains one or more types of dinucleotide step that can act as what we can term a ‘twist capacitor’ by making disproportionate contributions to absorbing twisting stress and thus having slopes (see Figure 3) that are steeper than expected given the imposed change of twist. Second, the presence of these ‘twist capacitors’ implies that at least some of the remaining steps can have smaller slopes and stay closer to their relaxed state. In the oligomers studied here, ACGT and AGCT use TpA steps to absorb the majority of the twist restraint, while ACGA and CCGA use CpG steps. Which YpR step comes into play is determined by how strongly its flanking base pairs limit its capacity to move between low and high twist substates. Thus the sequence environment and not just the presence of a given base pair step will influence the local mechanical properties of DNA.

**Figure 5.**
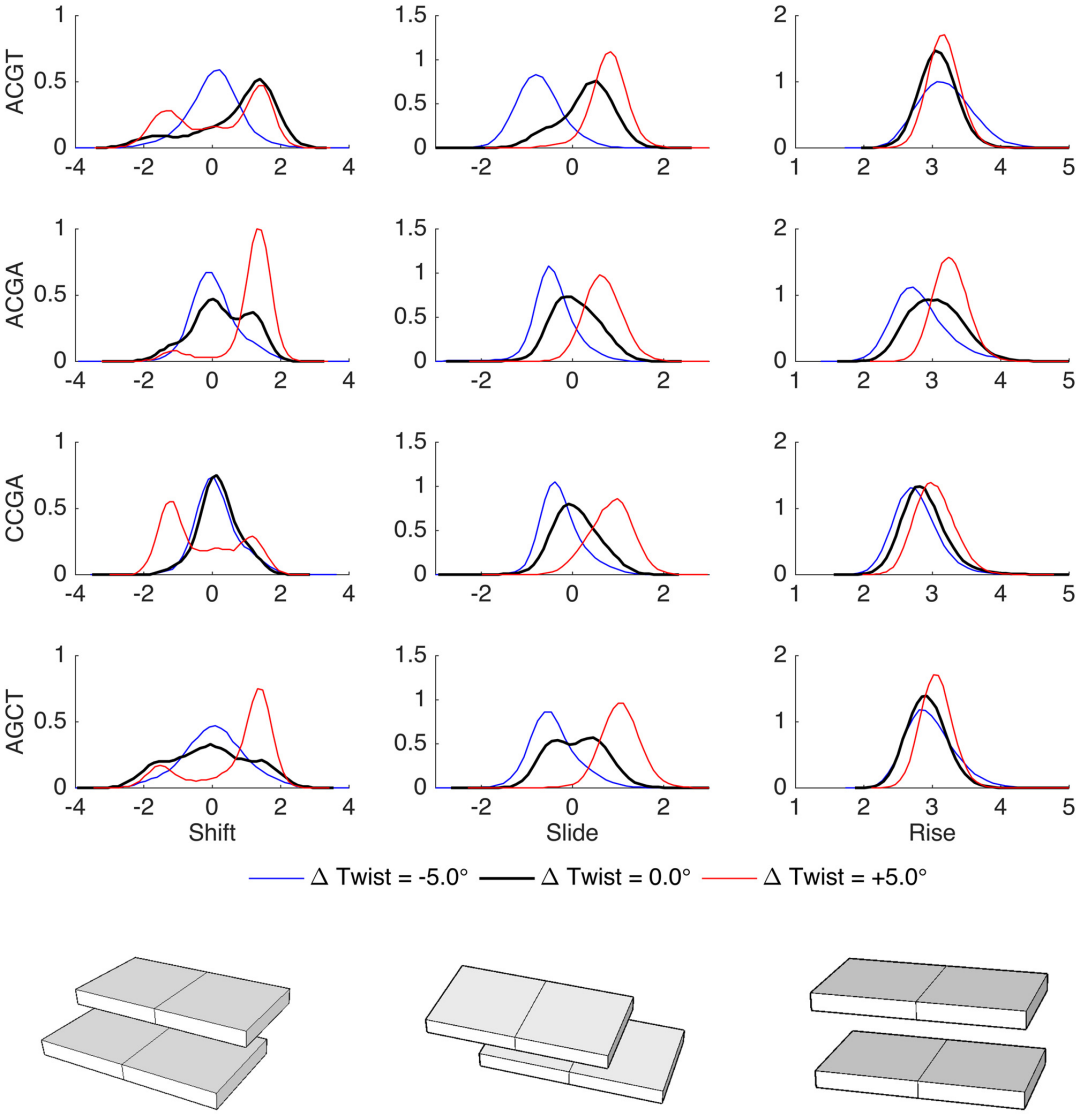
Normalized distributions of translational inter-base pair helical variables for the steps most affected by the imposed twist in each oligomer: TpA in ACGT and AGCT, CpG in ACGA and CCGA.

**Figure 6.**
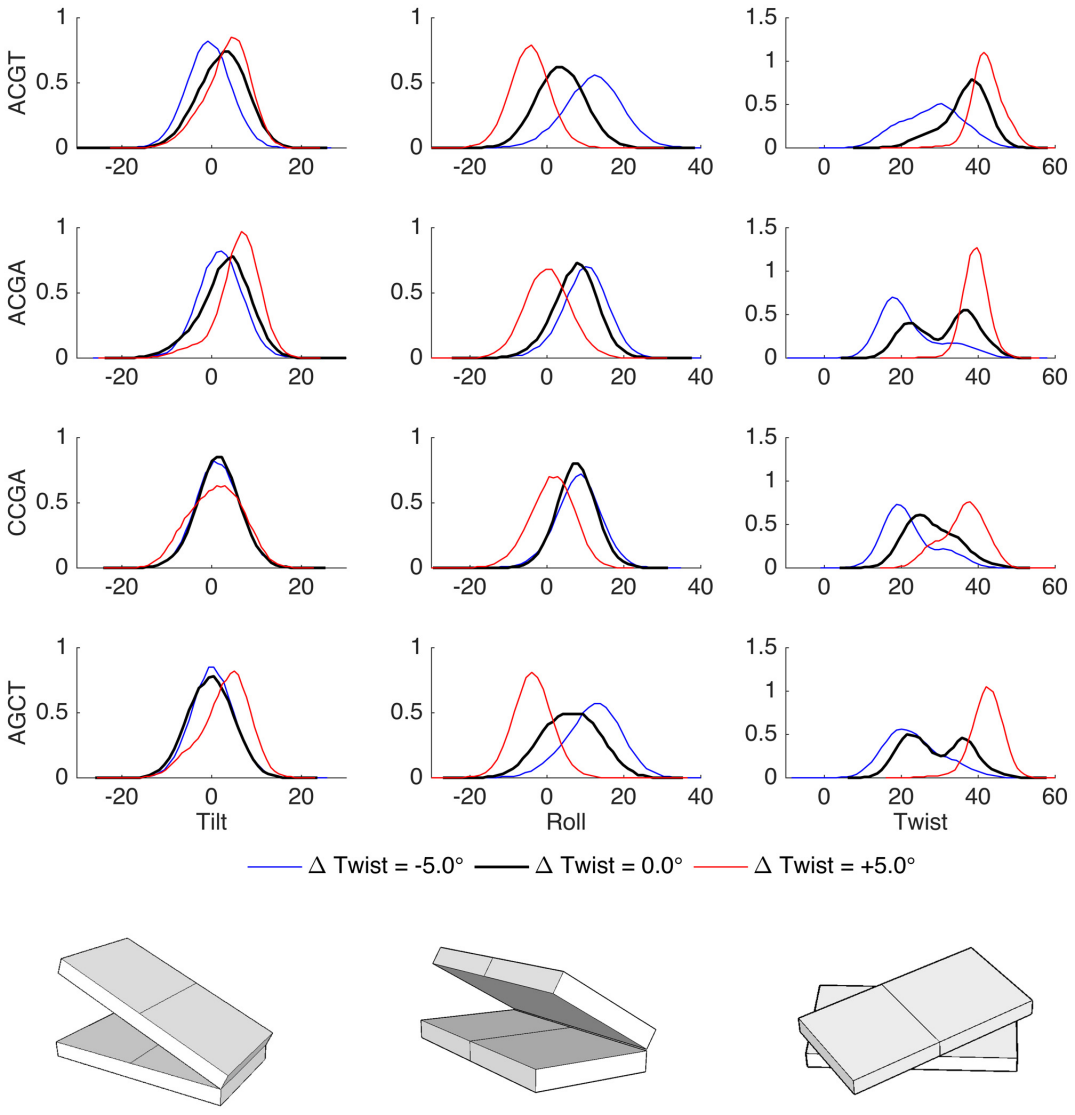
Normalized distributions of rotational inter-base pair helical variables for the steps most affected by the imposed twist in each oligomer: TpA in ACGT and AGCT, CpG in ACGA and CCGA.

Given that twist restraints on DNA lead not only to sequence-dependent changes in stiffness, but also to heterogeneous changes in local twisting, we can ask whether other helical parameters respond in a coupled fashion. The answer is shown in Figures 5 and 6 for translational and rotational parameters respectively. From the translational data, we see that rise is only affected when CpG steps dominate the absorption of twist, overtwisting leading to an increase in rise of up to 1 Å (which may explain the increased resistance to overtwisting in these cases since increased rise decreases the favorable stacking between base pairs). In contrast, slide is coupled to twist for all the oligomers studied, moving from negative to positive values and covering a range of roughly 2 Å. Lastly, shift shows a more subtle behavior, bimodality appears for some steps when twist is relaxed, but it is most pronounced in the overtwisted oligomers where both TpA and CpG steps show pronounced bimodality covering a range of 4 Å in shift. Shift transitions are strongly coupled to backbone conformations of the YpR junctions: BI Watson (5′ → 3′ stand) combined with BII Crick (3′ → 5′ strand) leading to positive shift and the reverse leading to negative shift. Passing to the rotational parameters (Figure 6), we see that roll decreases uniformly as twist increases, while tilt hardly affected by changes in twist.

## CONCLUSION

We have developed a new structural restraint that is able to accurately control the total twist of a segment of B-DNA during molecule dynamics simulations. This restraint has been used to study how four oligomeric base sequences react to over- or undertwisting. By using microsecond-scale simulations, we have been able to show that while the overall torsional moduli of the DNA oligomers we study are only weakly sequence-dependent, the double helix actually deforms in a very heterogeneous manner on the base pair level. Certain base pair steps, mainly pyrimidine-p-purine (YpR), but also purine-p-purine (RpR), are able to absorb a large part of the over- and undertwisting and, to use an electronic analogy, can be thought of as ‘twist capacitors’. Sufficiently long simulations were necessary to show that the molecular mechanism involved in this behavior is founded on backbone transitions of the ϵ (C3′-O3′) and ζ (O3′-P) dihedrals between the so-called BI and BII states, which can respectively favor high and low twist states. Preferences for one twist state, or for oscillation between two states, were already observed in unrestrained simulations of DNA (27,28), as was the significant role played by the base pairs flanking a given base pair step. Here, we show that the impact of these intrinsic base sequence effects are strikingly magnified in torsionally restrained DNA and is coupled with significant changes in helical parameters other than twist. Note that this result contrasts with the earlier findings of Kannan *et al.* (21), where all YpR steps seem to respond equally well to under- and overtwisting. This difference can mainly be attributed to the length of the simulations carried out in our study that were roughly 100× longer, and no longer hindered by problems with the backbone parameterization of earlier force fields. Our simulations enable the relatively slow equilibration of BI/BII states to occur. The fact that these states are particularly sensitive to the base pairs flanking a given dinucleotide step explains why certain YpR steps become unable to respond to torsional stress (such as the CpG step in the ACGT oligomer), or may only be able to efficiently absorb undertwisting (CpG in ACGA), or overtwisting (CpG in CCGA).

The fact that the behavior of a given base pair step under torsional stress is influenced by its sequence environment has recently been illustrated by the structural analysis of a 2D triangular self-assembled matrix of DNA, where one branch was constructed with one less base pair than would be necessary assuming a canonical B-DNA conformation (PDB ID: 5EOS) (47). The resulting imposed underwinding, of 4° per base pair step on average, was absorbed in a very heterogeneous way with two YpR steps being strongly underwound (TpG 25.7° and CpG 16.1°), while a third maintained a normal twist (TpG 36.7°). While the underwound steps lie in RYpRR environments (GTpGA and ACpGG, similar to the ACpGA step in our second oligomer which also becomes strongly undertwisted), the third is in a different YYpRY environment (CTpGT).

What are the possible consequences of these findings? First, we recall that several recent studies have shown that supercoiling density can vary significantly along genomic DNA and also vary rapidly with time (3). Although writhing generally dominates changes in twist on a large scale, local restraints that affect DNA segments up to ∼100 bp can favor changes in twist (48,49). Such regions notably can occur during abortive transcription events (50,51). When this occurs, our results suggest that the conformational changes will be very heterogeneous. This will have consequences for the mesoscopic conformation of DNA, as in the case of the formation of kinks (26,52) and can also impact interactions with proteins and protein complexes (5,53) whose binding strategy often relies on a pre-existing DNA conformation or specific local mechanical properties (54,55). It could thus constitute a useful biological strategy for inducing precise local changes using a simple mesoscopic restraint.

## DATA AVAILABILITY

DNA twisting restraint is an open source code available in the GitHub repository (https://github.com/annareym/PLUMED_DNA-Twist).

## Supplementary Material

Supplementary Data
