# Supplementary material for: Sequence-dependent response of DNA to torsional stress: a potential biological regulation mechanism
Source: Nucleic Acids Res. 2017 Dec 18;46(4):1684–94. doi: 10.1093/nar/gkx1270 (PMC5829783; doi:10.1093/nar/gkx1270)
Supplement: Supplementary Data [file gkx1270_supp.pdf]

SUPPLEMENTARY INFORMATION

Sequence-dependent response of DNA to torsional stress: a potential biological regulation mechanism

Anna Reymer[1,2*], Krystyna Zakrzewska[2] and Richard Lavery[2]

[1] Department of Chemistry and Molecular Biology, University of Gothenburg, Gothenburg, 40530, Sweden
[2] Institut de Biologie et Chimie des Protéines, Univ. Lyon I/CNRS UMR 5086, Lyon, 69367, France
* To whom correspondence should be addressed. Tel: +46 - (0) 31-786 9117; Email: anna.reymer@gu.se

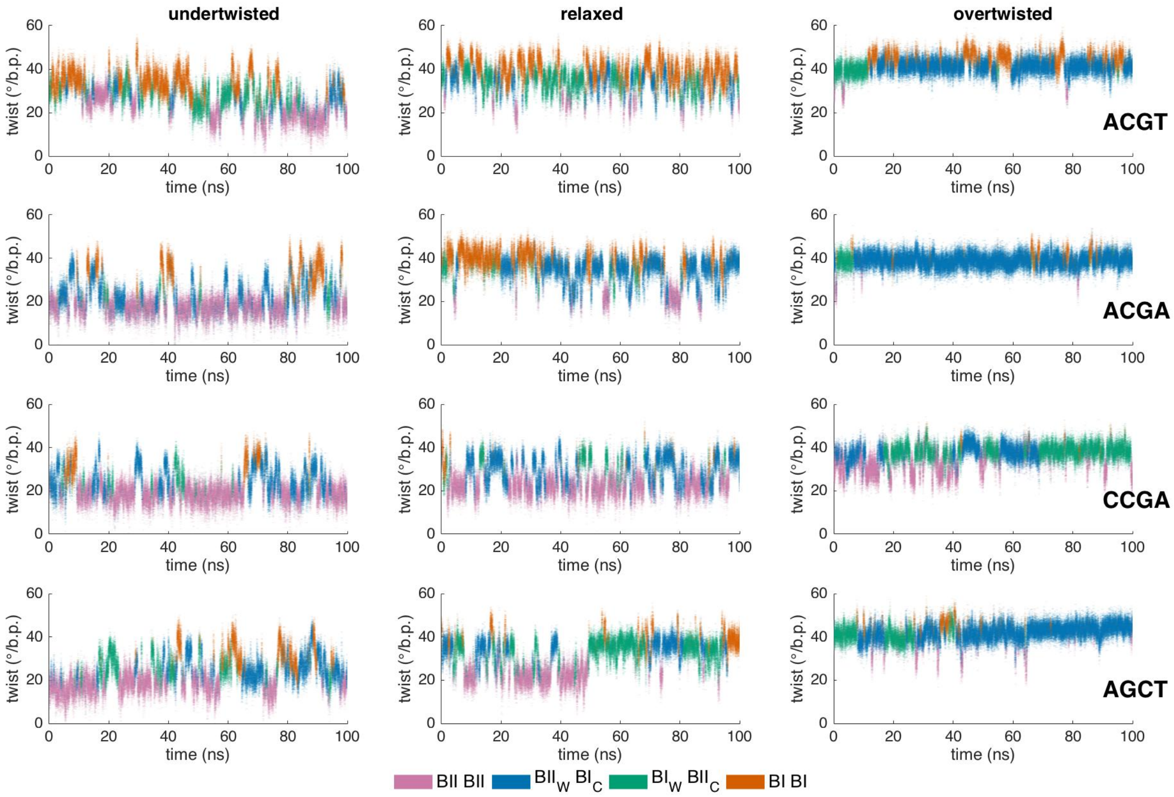

Figure S1. Time evolution of twist for the steps most affected by the imposed twist in each oligomer: TpA in ACGT and AGCT, CpG in ACGA and CCGA. The central panels show the behaviour of these steps in the relaxed oligomer, while the left and right hand panels show the impact of undertwisting (-5°) or overtwisting (+5°) with respect to the average base pair step twist of the restrained segments (34.9°). The time series are coloured as a function of the BI/BII state of the 3'-flanking phosphate junctions for the Watson (w) and Crick (c) strands.

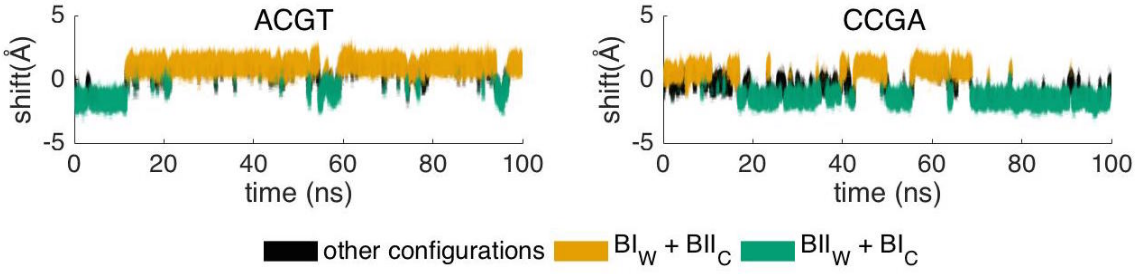

Figure S2. Time evolution of shift for TpA and CpG dinucleotide steps during the simulations of the ACGT and CCGA oligomers. For overtwisted DNA (+5.0° with respect to the average twist per base pair step for the restrained segments, 34.9°), these YpR steps show a bimodality in shift (see Figure S2). Changes in shift are coupled to BI/BII transitions in the YpR junctions: BI Watson (i.e. 5' → 3' strand) and BII Crick (i.e. 3' → 5' strand) leading to positive shift (coloured yellow above), while the reverse leads to negative shift (coloured green above).
